# Chemical components of *Dysoxylum densiflorum*

**DOI:** 10.1007/s13659-013-0025-8

**Published:** 2013-04-19

**Authors:** Ji Gu, Sheng-Yan Qian, Gui-Guang Cheng, Yan Li, Ya-Ping Liu, Xiao-Dong Luo

**Affiliations:** 125 State Key Laboratory of Phytochemistry and Plant Resources in West China, Kunming Institute of Botany, Chinese Academy of Sciences, Kunming, 650201 China; 225 University of Chinese Academy of Sciences, Beijing, 100049 China

**Keywords:** halimane, labdane, *Dysoxylum densiflorum*

## Abstract

Three new diterpenoids, including two halimanes, 5(10),13*E*-halimadiene-3*α*,15-diol (**1**), and 5(10),14-halimadiene-3*α*,13*ξ*-diol (**2**), one labdane, 12-(3-methyl-furan)-labd-8(17)-en-19-oic acid (**3**), together with sixteen known compounds were isolated from the barks of *Dysoxylum densiflorum*. All compounds were elucidated by extensive spectroscopic analysis.

**Electronic Supplementary Material:**

Supplementary material is available for this article at 10.1007/s13659-013-0025-8 and is accessible for authorized users.

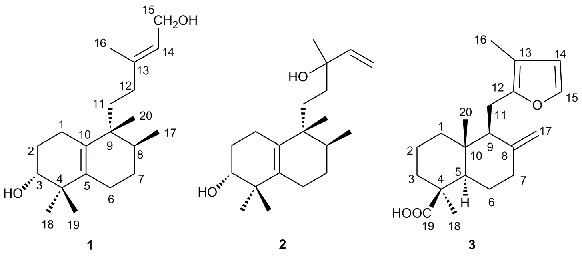

## Introduction

The plants of genus *Dysoxylum*, with about 200 species, is distributed naturally in India and south-east Asia. About 10 species of this genus have been found in Yunnan province.[1] According to the literatures, this genus have provided sorts of compounds, such as limonoids,[1,2] steroids,[3] sesquiterpenoids,[4] diterpenes,[5] triterpenes,[6] triterpene glycosides,[6d] and alkaloids.[7] Many plants of this genus have been used as traditional medicine by the indigenous.[8] *D. densiflorum,* is mainly distributed in southern China, Malaysia, and Philippines. Phytochemical research on *D. densiflorum* led to the isolation of terpenoids, steroids and flavonoids.[9] In the course of our ongoing investigation of genus *Dysoxylum* provided a series of bioactive chemical constituents by our lab,[1,2c,6b,10] including nineteen compounds from the EtOAc extracts of *D. densiflorum.* In the present research, three new diterpenoids including two new halimanes, 5(10),13*E*-halimadiene-3*α*,15-diol (**1**) and 5(10),14-halimadiene-3*α*,13*ξ*-diol (**2**), one new labdane, 12-(3-methyl-furan)-labd-8(17)-en-19-oic acid (**3**), were isolated and characterized by extensive spectroscopic analysis. Compounds **1** and **2** possessed 5(10)-halimane skeleton were rare in nature since the 20-methyl rearranged labdane skeleton does not conform to the biogenetic 'isoprene rule'.[11] The known compounds were determined as piscidinol A,[12] 3-oxotirucalla-7,24-dien-23-ol,[13] 3*β*-acetoxy-betulin,[14] isocupressic acid,[15] 12-oxo-15-hydroxylabda-8(17),13*E*-dien-19-oic acid,[16] 14(*R*),15-dihydroxy-8(17),12(*E*)-labdadien-19-oic acid,[17] 15-nor-14-oxolabda-8(17),12*E*-dien-19-oic acid,[18] cryptotrienolic acid,[19] 7-hydroxy-cupressic acid,[20] (+)-labda-8(17),13(Z)-dien-15,16-diol,[21] 4*β*-hydroxy-15-(3-methyl-2-butenyl)-aromadendr-$\Delta^{10(12)}$-en,[22] 4(15)-eudesmene-1*β*,7*α*-diol,[23] *β*-sitosterol, 7*α*-hydroxysitosterol,[24] 3,4,5-trihydroxycinnamate,[25] and 5,6-dihydroxy-6-methyl-3-en-2-one.[26] Herein, we report the isolation and structural elucidation of the isolated compounds.

**1** **2** **3**

## Results and Discussion

Compound **1**, as an optically active white amorphous powder {$[\alpha]^{19}_{D}$ + 68.3 (*c* 0.172, $Me_2CO$)}, possessed the molecular formula $C_{20}H_{34}O_2$ by HREIMS at *m/z* 306.2566 $[M]^+$, indicating four degrees of unsaturation. The IR spectrum revealed absorption bands for hydroxyl (3430 $cm^{-1}$) and olefinic bond (1631 $cm^{-1}$). The $^1H$, $^{13}C$ NMR and DEPT spectra of **1** exhibited 20 carbon resonances, assigned to one tetrasubstituted double bond [$\delta_C$ 132.4 (s), 138.0 (s)]; one trisubstituted double bond [$\delta_C$ 125.5 (d), 138.4 (s)] with a corresponding proton at $\delta_H$ 5.33 (t, *J* = 6.2 Hz, H-14); five methyls with corresponding four tertiary methyl protons at $\delta_H$ 1.62 (Me-16), 1.04 (Me-18), 0.94 (Me-19), and 0.82 (Me-20), and a secondary methyl protons at $\delta_H$ 0.85 (d, *J* = 6.9 Hz, Me-17); seven methylenes (one oxygenated); two methines (one oxygenated); and two quaternary carbons. Besides a

*To whom correspondence should be addressed. E-mail: xdluo@mail.kib.ac.cn

**Table 1.** $^{1}$**H NMR data of 1–3**$^{a}$ **(δ in ppm and J in Hz)**

| no. | 1 | 2 | 3 |
|---|---|---|---|
| 1a | 2.15, m | 2.06, overlap | 1.89, m |
| 1b | 1.95, overlap | 1.99, overlap | 1.25, m |
| 2a | 1.71, m | 1.67, m | 1.92, overlap |
| 2b | 1.59, m | 1.56, m | 1.49, overlap |
| 3a | 3.40, dd (7.5, 4.1) | 3.36, m | 2.14, m |
| 3b | | | 1.11, td (13.2, 3.9 ) |
| 5 | | | 1.46, m overlap |
| 6a | 2.05, overlap | 2.04, overlap | 2.00, m |
| 6b | 2.00, overlap | 1.96, overlap | 1.91, overlap |
| 7a | 1.46, overlap | 1.43, overlap | 2.33, overlap |
| 7b | 1.36, m | 1.34, m | 1.93, overlap |
| 8 | 1.63, overlap | 1.61, overlap | |
| 9 | | | 2.31, overlap |
| 10 | | | |
| 11a | 1.48, overlap | 1.42, overlap | 2.75, dd (15.4, 2.9) |
| 11b | 1.44, overlap | 1.39, overlap | 2.67, d (10.4) |
| 12a | 1.92, overlap | 1.42, overlap | |
| 12b | 1.63, overlap | 1.13, m | |
| 14 | 5.33, t (6.2) | 5.90, dd (17.3, 10.7) | 6.12, d (1.6) |
| 15a | 4.04, t (5.7) | 5.18, dd (17.3, 1.8) | 7.22, d (1.6) |
| 15b | | 4.94, dd (10.7, 1.8) | |
| 16 | 1.62, s | 1.20, s | 1.94, s |
| 17a | 0.85, d (6.9) | 0.82, d (7.2) | 4.75, s |
| 17b | | | 4.60, s |
| 18 | 1.04, s | 1.01, s | 1.22, s |
| 19 | 0.94, s | 0.92, s | |
| 20 | 0.82, s | 0.81, s | 0.72, s |

$^{a}$Compounds **1**–**3** were measured in acetone-$d_6$.

**Table 2.** $^{13}$**C NMR data of 1–3**$^{a}$ **(δ in ppm and J in Hz)**

| no. | 1 | 2 | 3 |
|---|---|---|---|
| 1 | 25.3 $CH_2$ | 25.1 $CH_2$ | 39.8 $CH_2$ |
| 2 | 28.6 $CH_2$ | 28.6 $CH_2$ | 20.8 $CH_2$ |
| 3 | 75.9 CH | 75.9 CH | 38.9 $CH_2$ |
| 4 | 40.7 C | 40.6 C | 44.5 C |
| 5 | 138.0 C | 137.5 C | 56.6 CH |
| 6 | 26.5 $CH_2$ | 26.6 $CH_2$ | 26.9 $CH_2$ |
| 7 | 28.1 $CH_2$ | 28.1 $CH_2$ | 39.2 $CH_2$ |
| 8 | 34.2 CH | 34.2 CH | 149.0 C |
| 9 | 41.2 C | 40.8 C | 54.6 CH |
| 10 | 132.4 C | 132.7 C | 41.0 C |
| 11 | 35.2 $CH_2$ | 30.4 $CH_2$ | 22.2 $CH_2$ |
| 12 | 34.8 $CH_2$ | 37.4 $CH_2$ | 151.4 C |
| 13 | 138.4 C | 72.8 C | 114.0 C |
| 14 | 125.5 CH | 147.2 CH | 113.6 CH |
| 15 | 59.1 $CH_2$ | 111.1 $CH_2$ | 140.2 CH |
| 16 | 16.3 $CH_3$ | 28.1 $CH_3$ | 10.1 $CH_3$ |
| 17 | 16.4 $CH_3$ | 16.3 $CH_3$ | 107.3 $CH_2$ |
| 18 | 25.5 $CH_3$ | 25.4 $CH_3$ | 29.3 $CH_3$ |
| 19 | 20.4 $CH_3$ | 20.4 $CH_3$ | 178.7 C |
| 20 | 21.3 $CH_3$ | 21.6 $CH_3$ | 13.0 $CH_3$ |

$^{a}$Compounds **1**–**3** were measured in acetone-$d_6$.

tetrasubstituted double bond and a trisubstituted double bond, the degrees of unsaturation required two rings for the structure. Similarities of the chemical shifts and coupling constants of **1** with known compound 3ξ-hydroxy-5(10),13*E*-halimadien-15-al[11b] revealed that **1** possesses a halimane-type diterpenoid skeleton (Tables 1 and 2). The difference was the presence of an oxygenated methylene in **1** with the chemical shift of $\delta_C$ 59.1 (t), instead of an aldehyde group in 3ξ-hydroxy-5(10),13*E*-halimadien-15-al [$\delta_C$ 189.8 (d)] at C-15. This was further confirmed by the HMBC correlation from $\delta_H$ 5.33 (t, $J$ = 6.2 Hz, H-14) and 3.47 (15-OH) to $\delta_C$ 59.1 (t, C-15), and from $\delta_H$ 4.04 (t, $J$ = 5.7 Hz, H-15) to $\delta_C$ 138.4 (s, C-13).

According to literatures,[11c,11h,11j] the Me-20 of **1** was positioned at axial bond for reducing steric hindrance of C-9 side chain, and assigned as β-orientation temporarily. In the ROESY spectrum of **1**, ROESY correlations of $\delta_H$ 0.82 (Me-20)/2.15 (H-1a) assigned the β-position of H-1a. Furthermore, cross-peaks of 1.95 (H-1b)/3.59 (3-OH), 3.59 (3-OH)/1.04 (Me-18), 1.04 (Me-18)/2.00 (H-6b), 2.00 (H-6b)/1.63 (H-8) suggested they were all located on the same face and assigned as α-position. Accordingly, the Me-17 and Me-19 were elucidated to be β-oriented. In addition, ROESY correlation of $\delta_H$ 4.04 (H-15)/1.62 (Me-16) indicated an *E*-configuration of $\Delta^{13/14}$. Thus, compound **1** was elucidated to be 5(10),13*E*-halimadiene-3α,15-diol as shown in Figure 1.

Compound **2** had an identical molecular formula $C_{20}H_{34}O_2$ as **1** according to its HREIMS at $m/z$ 306.2550 $[M]^+$. The $^{1}$H and $^{13}$C NMR spectra of **2** (Tables 1 and 2) were close similarities to those of **1**, except that the allylic alcohol moiety ($R_2C$=$CHCH_2OH$, C-13–C-16) in **1** was changed to be an oxygenated quaternary carbon ($\delta_C$ 72.8) at C-13 and a terminal double bond [$\delta_C$ 147.2 (d), 111.1 (t)] at $\Delta^{14/15}$ in **2**. The assumption was further supported by the HMBC correlations of $\delta_H$ 5.18 (dd, $J$ = 17.3, 1.8 Hz, H-15a) and 4.94 (dd, $J$ = 10.7, 1.8 Hz, H-15b) with $\delta_C$ 72.8 (s, C-13), of $\delta_H$ 1.13 (m, H-12b) and 1.20 (s, Me-16) with $\delta_C$ 147.2 (d, C-14). Other parts of **2** were identical to those of **1**, as supported by HSQC, HMBC, and ROESY spectra. Thus, **2** was deduced as 5(10),14-halimadiene-3α,13ξ-diol.

**Figure 1.** Selected HMBC ( ) and ROESY ( ) correlations of **1**

Compound **3**, as a white amorphous powder, exhibited the molecular formula $C_{20}H_{28}O_3$ by HREIMS at $m/z$ 316.2039 $[M]^+$, indicating seven degrees of unsaturation. The $^{1}$H, $^{13}$C NMR and DEPT spectra of **3** (Tables 1 and 2) exhibited 20 carbon signals, ascribed to one carbonyl group [$\delta_C$ 178.7 (s)]; one exocylic double bond [$\delta_C$ 149.0 (s), 107.3 (t)], with corresponding protons at $\delta_H$ 4.75, 4.60 (s, H-17); four olefinic carbons [$\delta_C$ 113.6 (d), 114.0 (s), 140.2 (d), 151.4 (s)], with corresponding protons at $\delta_H$ 6.12 (d, $J$ = 1.6 Hz, H-14) and 7.22 (d, $J$ = 1.6 Hz, H-15); three tertiary methyls with corresponding methyl protons at $\delta_H$ 1.94 (Me-16), 1.22 (Me-18), and 0.72 (Me-20); six methylenes, two methines, and two quaternary carbons. As four degrees of unsaturation were accounted by one carbonyl group and three C-C double bonds, the remaining three degrees of unsaturation were attributed to three rings for **3**. Comparison of the $^{1}$H and $^{13}$C NMR data of **3** with those of 12-oxo-15-hydroxylabda-8(17),13*E*-dien-19-oic acid[16] suggested that **3** possesses a labdane skeleton. A furan moiety was suggested on the basis of four olefinic carbons at $\delta_C$ 113.6 (d), 114.0 (s), 140.2 (d), 151.4 (s), and the corresponding protons at $\delta_H$ 6.12 (d, $J$ = 1.6 Hz, H-14) and 7.22 (d, $J$ = 1.6 Hz, H-15). The HMBC correlations of $\delta_H$ 2.31 (overlap, H-9), 1.94 (s, Me-16), 6.12 (d, $J$ = 1.6Hz, H-14) and 7.22 (d, $J$ = 1.6Hz, H-15) with $\delta_C$ 151.4 (s, C-12), of $\delta_H$ 2.75

(dd, $J$ = 15.4, 2.9 Hz, H-11a), 2.67 (d, $J$ = 10.4 Hz, H-11b) with $\delta_C$ 114.0 (s, C-13), and of $\delta_H$ 1.94 (s, Me-16) with $\delta_C$ 113.6 (d, C-14) further permitted the assignment of a 3-methyl-furan moiety at C-12.

In the ROESY spectrum of **3**, ROESY correlations of $\delta_H$ 1.46 (H-5)/1.25 (H-1b), 2.31 (H-9) suggested that they all located on the same side. Me-20 ($\delta_H$ 0.72) did not show ROESY correlation to any of above protons, but showed correlations with the proton signals at $\delta_H$ 2.75, 2.67 (2H, H-11) and 1.89 (H-1a), which placed them at another side. The A/B ring was deduced to be *trans*-fused, which was consistent with labdane-type diterpenoids reported.[15,16,18,27] According to the literatures, H-5 was assigned at $\alpha$-position, which further assigned the H-9 to be $\alpha$-oriented and Me-20 to be $\beta$-oriented. Moreover, ROESY cross-peak of $\delta_H$ 1.46 (H-5)/1.22 (Me-18) suggested the $\alpha$-orientation of Me-18. Hence, compound **3** was established as 12-(3-methyl-furan)-labd-8(17)-en-19-oic acid (Figure 2).

**Figure 2.** Selected HMBC ( ) and ROESY ( )

## Experimental Section

**General Experimental Procedures.** Optical rotations were obtained with a Jasco P-1020 Automatic Digital Polariscope. UV spectrua was measured with a Shimadzu UV2401PC in MeOH solution. IR spectra (KBr) were obtained on a Bruker tensor-27 infrared spectrophotometer. $^1$H, $^{13}$C, and 2D NMR spectra were recorded on a Bruker AM-400, a DRX-500 NMR and an Avance III 600 spectrometer with TMS as internal standard. MS data were obtained on a Waters Autospec Premier P776 for HREI. Column chromatography (CC) was performed on Silica gel (200–300 mesh, Qingdao Marine Chemical Co., Ltd., Qingdao, China) and RP-18 gel (20–45 $\mu$m, Fuji Silysia Chemical Co., Ltd., Tokyo, Japan). Fractions were monitored by TLC (GF 254, Qingdao Marine Chemical Co., Ltd., Qingdao, China), and spots were visualized by 10% $H_2SO_4$-ethanol reagent.

**Plant Material.** The barks of *Dysoxylum densiflorum* was collected from Xishuangbanna Autonomous Prefecture, Yunnan Province, China, and identified by Jingyun Cui of Xishuangbanna Botanic Garden. A voucher specimen (Cui200811-18) has been deposited at Herbarium of Kunming Institute of Botany, Chinese Academy of Sciences.

**Extraction and Isolation.** The air-dried barks (5.0 kg) of *Dysoxylum densiflorum* was extracted with MeOH three times under normal temperature. After removal of the solvent, the extract suspended in $H_2O$ and extracted with ethyl acetate four times. The EtOAc fraction (129.0 g) was subjected to CC on Si gel, eluted with gradient mixtures of $CHCl_3$-$Me_2CO$ (1:0–0:1). According to differences in composition monitored by TLC, five fractions were obtained. Fraction III (15.6 g) was separated to MPLC with RP-18 CC (MeOH-$H_2O$, 3:7–8:2), then followed by Si gel CC (petroleum ether-EtOAc, 5:1–1:1) to afford **3** (5.0 mg), 3,4,5-trihydroxycinnamate (12.0 mg), and two subfractions IIIa and IIIb. Subfraction IIIa was chromatographed on a Si gel CC ($CHCl_3$-$Me_2CO$, 12:1–8:1) to yield 7-hydroxy-cupressic acid (9.0 mg) and 5,6-dihydroxy-6-methyl-3-en-2-one (6.0 mg). Subfraction IIIb was separated with a Si gel CC (petroleum ether-$Me_2CO$, 8:1–5:1) to get piscidinol A (47.0 mg), 4(15)-eudesmene-1$\beta$,7$\alpha$-diol (13.0 mg), and a mixture. The mixture was further chromatographed on a Si gel CC ($CHCl_3$-$Me_2CO$, 10:1–6:1) to yield isocupressic acid (20.0 mg). Fraction IV (5.3 g) was subjected to MPLC with RP-18 CC (MeOH-$H_2O$, 3:7–7:3) to afford a mixture. The mixture was lately separated by Si gel CC (petroleum ether-$Me_2CO$, 4:1–2:1) to yield 15-nor-14-oxolabda-8(17),12*E*-dien-19-oic acid (4.0 mg) and 3$\beta$-acetoxy-betulin (26.0 mg). Fraction V (24.0 g) was isolated with MPLC RP-18 CC (MeOH-$H_2O$, 2:8–7:3) to obtained different subfractions Va–c. Subfraction Va was chromatographed on a Si gel CC ($CHCl_3$-$Me_2CO$, 8:1–5:1) to yield **2** (33.0 mg) and 3-oxotirucalla-7,24-dien-23-ol (22.0 mg). Then, subfraction Vb was purified by a Si gel CC (petroleum ether-EtOAc, 3:1–2:1) to the isolation of **1** (18.0 mg), (+)-labda-8(17),13(*Z*)-dien-15,16-diol (7.0 mg), and cryptotrienolic acid (2.0 mg). With MeOH-$H_2O$ (4:6–6:4) as elution solvent, subfraction Vc was separated with RP-18 CC into two mixtures, one of which was further purified by a Si gel CC (petroleum ether-EtOAc, 1:1) to give the separation of 12-oxo-15-hydroxylabda-8(17),13*E*-dien-19-oic acid (14.0 mg), 7$\alpha$-hydroxysitosterol (24.0 mg), and $\beta$-sitosterol (425.0 mg). The other one was subjected through a Si gel CC (petroleum ether-$Me_2CO$, 3:1–2:1) to afford 4$\beta$-hydroxy-15-(3-methyl-2-butenyl)-aromadendr-$\Delta^{10(12)}$-en (14.0 mg) and 14(*R*),15-dihydroxy-8(17),12(*E*)-labdadien-19-oic-acid (26.0 mg).

**5(10),13*E*-halimadiene-3$\alpha$,15-diol (1):** a white amorphous powder; $[\alpha]_D^{19}$ + 68.3 ($c$ 0.172, $Me_2CO$); IR (KBr) $\nu_{max}$ 3430, 2965, 2931, 1631, 1467, 1381, 1050, 1006 cm$^1$; $^1$H (400 MHz) and $^{13}$C NMR (150 MHz) data ($Me_2CO$), see Tables 1 and 2, respectively; HREIMS $m/z$ 306.2566 (calcd for $C_{20}H_{34}O_2$ $[M]^+$, 306.2559).

**5(10),14-halimadiene-3$\alpha$,13$\xi$-diol (2):** a white amorphous powder; $[\alpha]_D^{20}$ + 61.0 ($c$ 0.120, $Me_2CO$); IR (KBr) $\nu_{max}$ 3431, 2965, 2925, 1634, 1457, 1380, 1049 cm$^{-1}$; $^1$H (400 MHz) and $^{13}$C NMR (150 MHz) data ($Me_2CO$), see Tables 1 and 2, respectively; HREIMS $m/z$ 306.2550 (calcd for $C_{20}H_{34}O_2$ $[M]^+$, 306.2559).

**12-(3-methyl-furan)-labd-8(17)-en-19-oic acid (3):** a white amorphous powder; $[\alpha]_D^{19}$ – 4.7 ($c$ 0.114, $Me_2CO$); UV (MeOH) $\lambda_{max}$ (log $\varepsilon$) 218 (3.03), 202 (3.16) nm; IR (KBr) $\nu_{max}$ 3440, 2958, 2934, 1693, 1632, 1449, 1266, 1181, 1151 cm$^{-1}$;

$^{1}H$ (500 MHz) and $^{13}C$ NMR (150 MHz) data ($Me_2CO$), see Tables 1 and 2, respectively; HREIMS *m/z* 316.2039 (calcd for $C_{20}H_{28}O_3$ $[M]^+$, 316.2038).

## Electronic Supplementary Material

Supplementary material is available in the online version of this article at http://dx.doi.org/ 10.1007/s13659-013-0025-8 and is accessible for authorized users.

## Acknowledgments

The authors are grateful to the Natural Science Foundation of China (81225024, 31170334, 21072198), the National Basic Research Program of China (973 Program 2009CB522300), for partly financial support, and to the analytical group of the Laboratory of Phytochemistry, Kunming Institute of Botany, for the spectral measurement.

**Open Access** This article is distributed under the terms of the Creative Commons Attribution License which permits any use, distribution, and reproduction in any medium, provided the original author(s) and source are credited.

## Electronic supplementary material

Supplementary material, approximately 797 KB.
