# Supplementary material for: Chemical components of Dysoxylum densiflorum
Source: Nat Prod Bioprospect. 2013 Apr 19;3(2):66–9. doi: 10.1007/s13659-013-0025-8 (PMC4131656; doi:10.1007/s13659-013-0025-8)
Supplement: Supplementary file 1 — Supplementary material, approximately 797 KB. [file 13659_2013_25_MOESM1_ESM.pdf]

Electronic Supplementary Material

Chemical components of *Dysoxylum densiflorum*

Ji GU,[a,b,†] Sheng-Yan QIAN,[a,b,†] Gui-Guang CHENG,[a,b] Yan LI,[a] Ya-Ping LIU,[a,]* and Xiao-Dong LUO[a,]*

[a]State Key Laboratory of Phytochemistry and Plant Resources in West China, Kunming Institute of Botany, Chinese Academy of Sciences, Kunming 650201, China

[b]University of Chinese Academy of Sciences, Beijing 100049, China

[†]These authors contributed equally to this work.

Received 18 March 2013; Accepted 6 April 2013

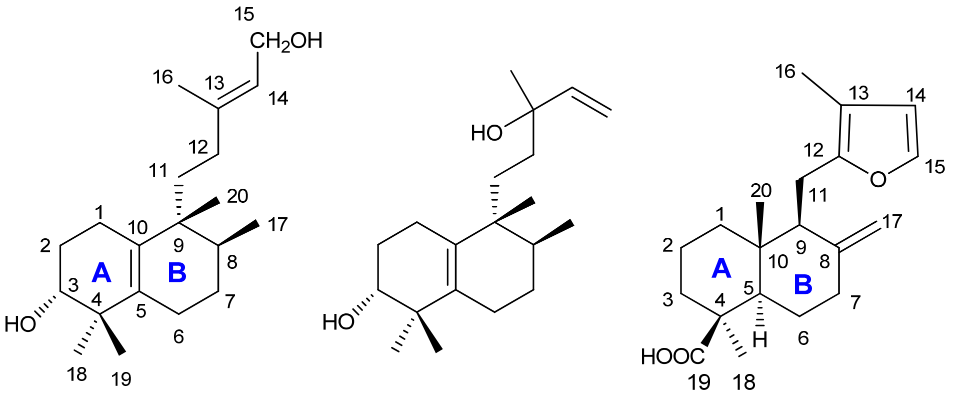

1 **2** **3**

Structures of compounds **1**–**3**

*To whom correspondence should be addressed. E-mail: xdluo@mail.kib.ac.cn

Content list

Figure 1S-6S. NMR and MS spectra of 5(10),13*E*-halimadiene-3α,15-diol (**1**)

Figure 7S-12S. NMR and MS spectra of 5(10),14-halimadiene-3α,13ξ-diol (**2**)

Figure 13S-18S. NMR and MS spectra of 12-(3-methyl-furan)-labd-8(17)-en-19-oic acid (**3**)

Figure 1S. ^{1}H NMR of 5(10), 13*E*-halimadiene-3*α*,15-diol (**1**)

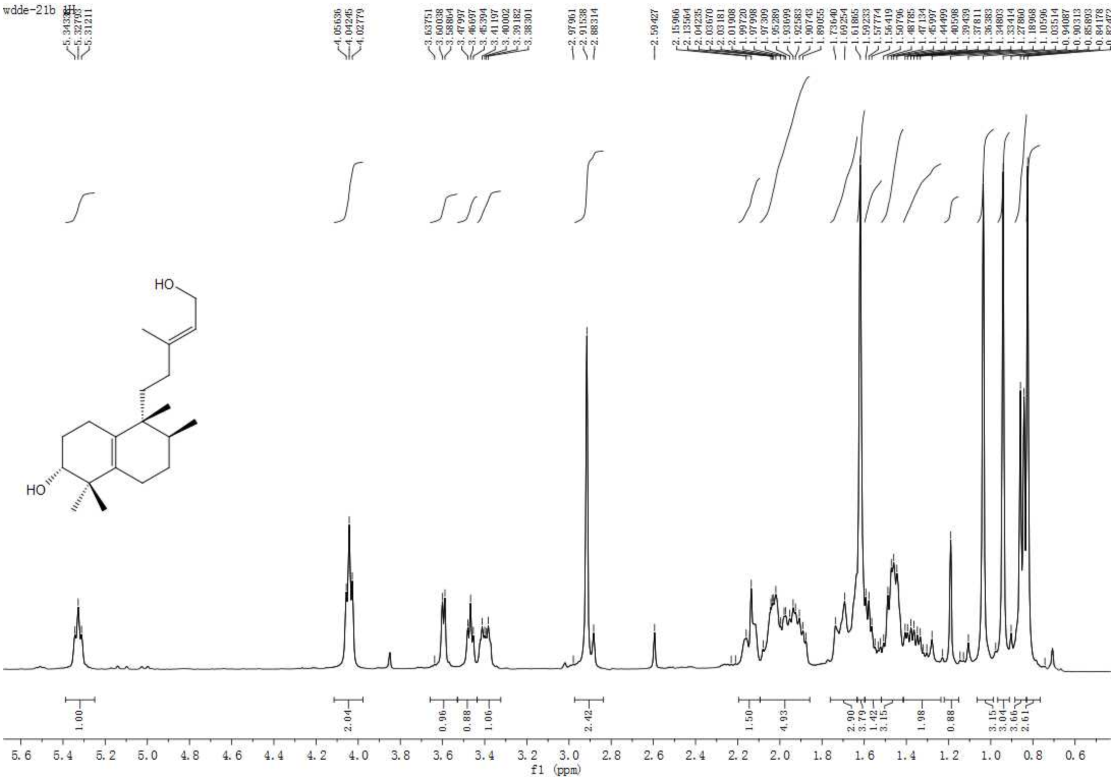

Figure 2S. ^{13}C NMR and DEPT of 5(10), 13*E*-halimadiene-3*α*,15-diol (**1**)

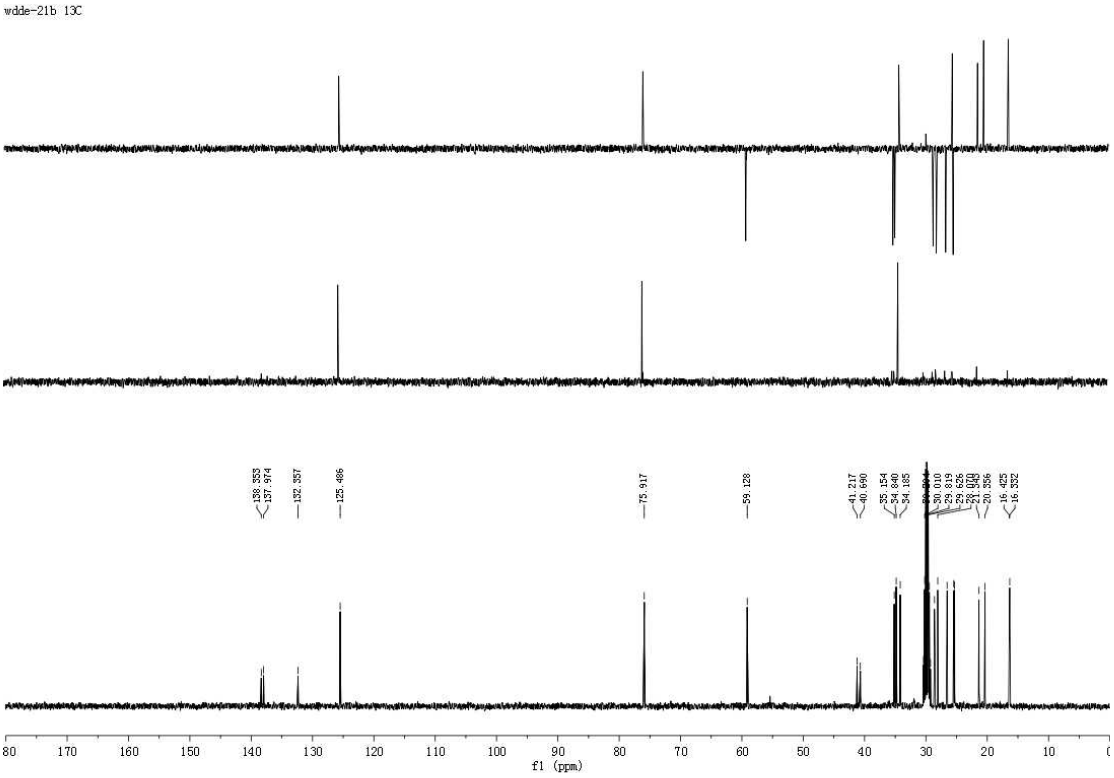

Figure 3S. HSQC of 5(10), 13*E*-halimadiene-3*α*,15-diol (**1**)

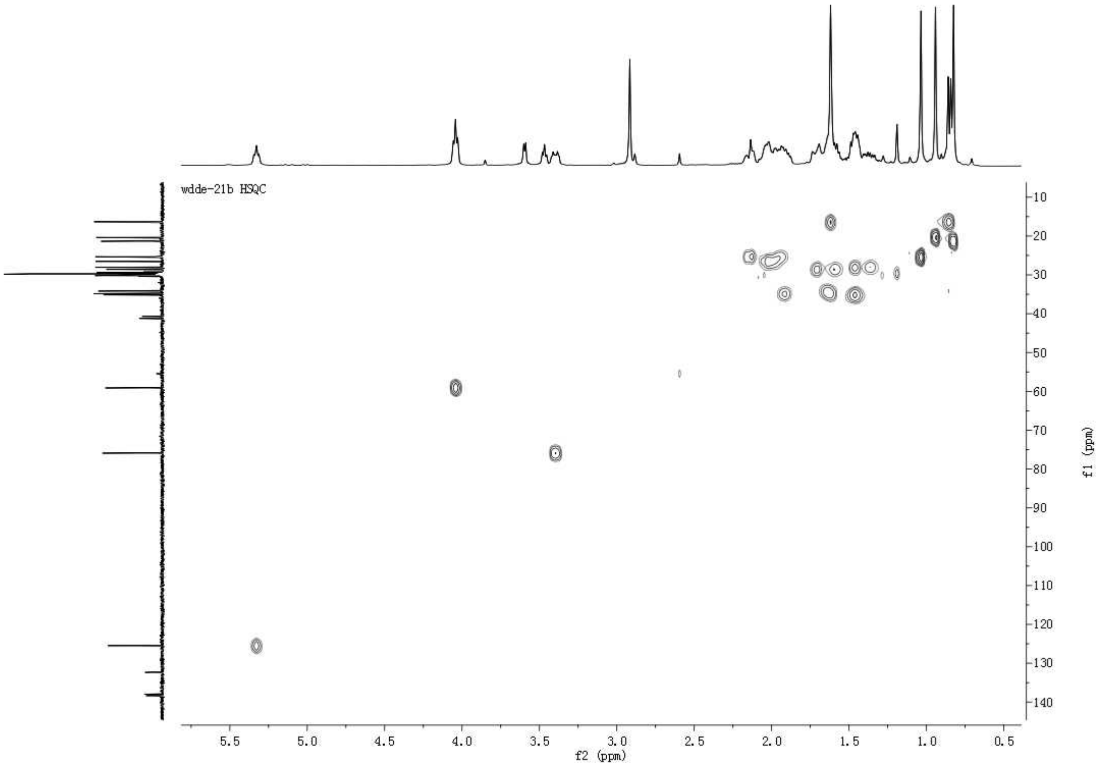

Figure 4S. HMBC of 5(10), 13*E*-halimadiene-3*α*,15-diol (**1**)

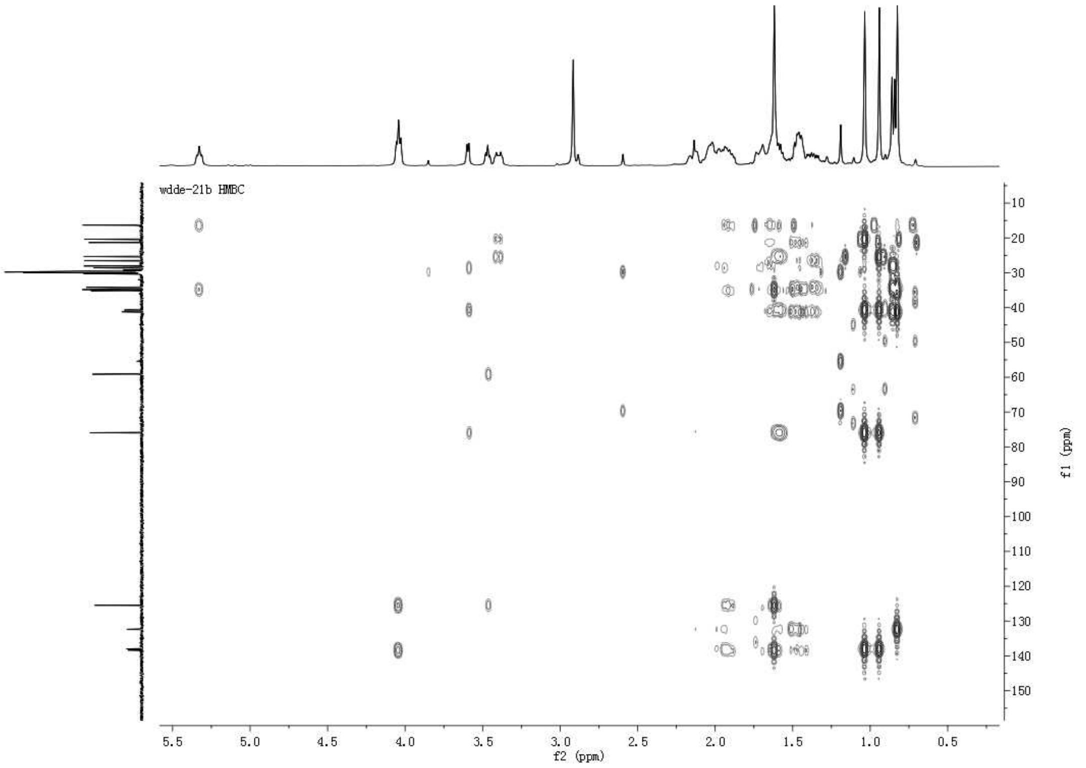

Figure 5S. ROESY of 5(10), 13*E*-halimadiene-3*α*,15-diol (**1**)

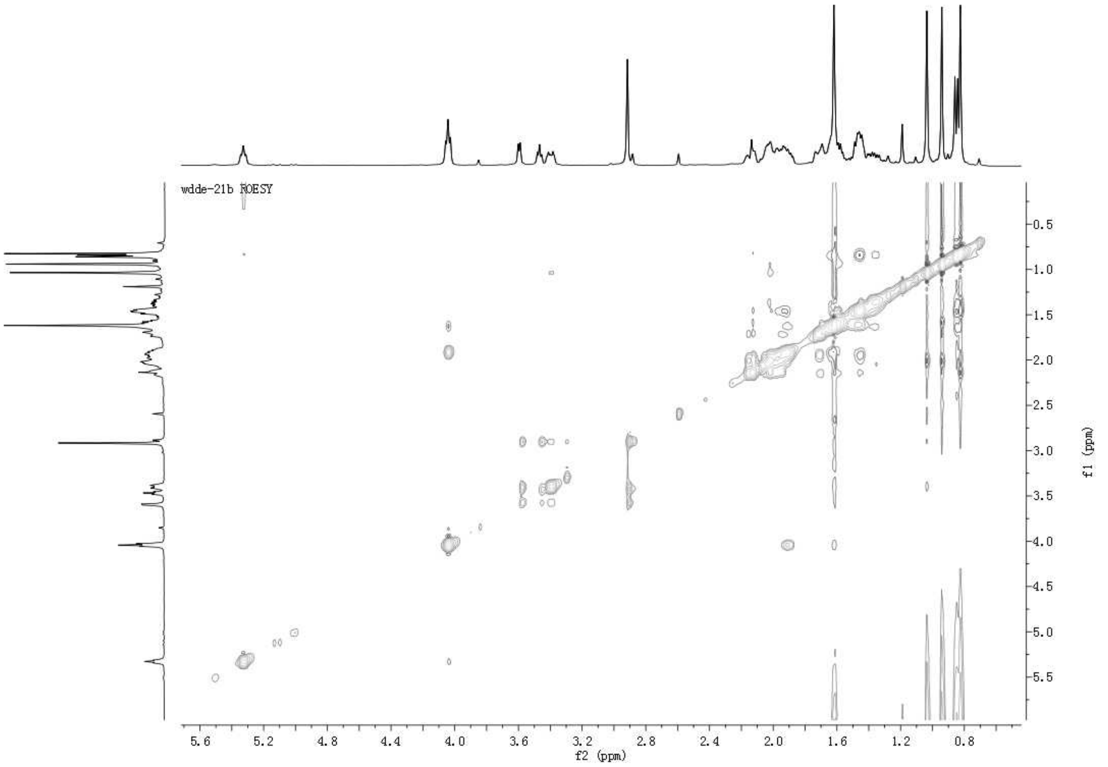

Figure 6S. HREIMS of 5(10), 13*E*-halimadiene-3*α*,15-diol (**1**)

Elemental Composition Report **Page 1**

Single Mass Analysis
Tolerance = 10.0 PPM / DBE: min = -10.0, max = 120.0
Selected filters: None

Monoisotopic Mass, Odd and Even Electron Ions
15 formula(e) evaluated with 1 results within limits (up to 51 closest results for each mass)
Elements Used:
C: 0-200 H: 0-400 O: 2-4

wdde21b
16:52:05 20-Nov-2012
Voltage EI+

KIB
M121122EA-10AFAMM 14 (1.286)
306.2566

Autospec Premier
P776
3.89

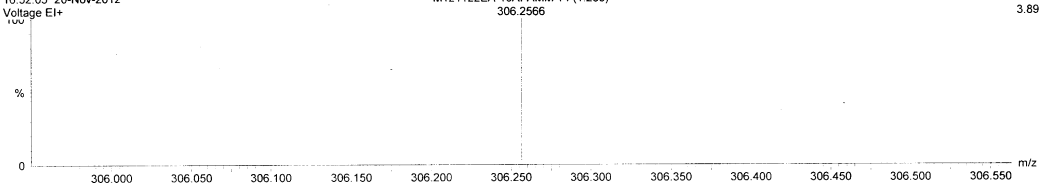

Minimum:				-10.0		
Maximum:		100.0	10.0	120.0		
Mass	Calc. Mass	mDa	PPM	DBE	i-FIT	Formula
306.2566	306.2559	0.7	2.3	4.0	5546025.5	C20 H34 O2

Figure 7S. ^{1}H NMR of 5(10),14-halimadiene-3*α,*13*ξ*-diol (**2**)

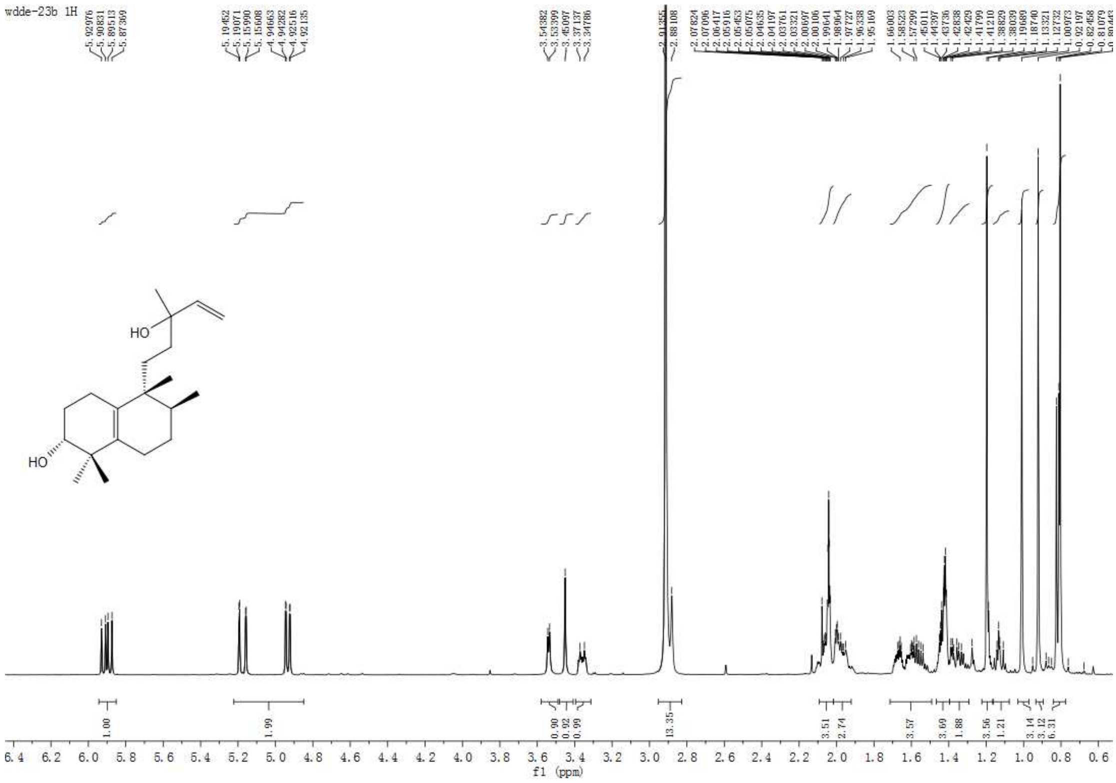

Figure 8S. ^{13}C NMR and DEPT of 5(10),14-halimadiene-3*α,*13*ξ*-diol (**2**)

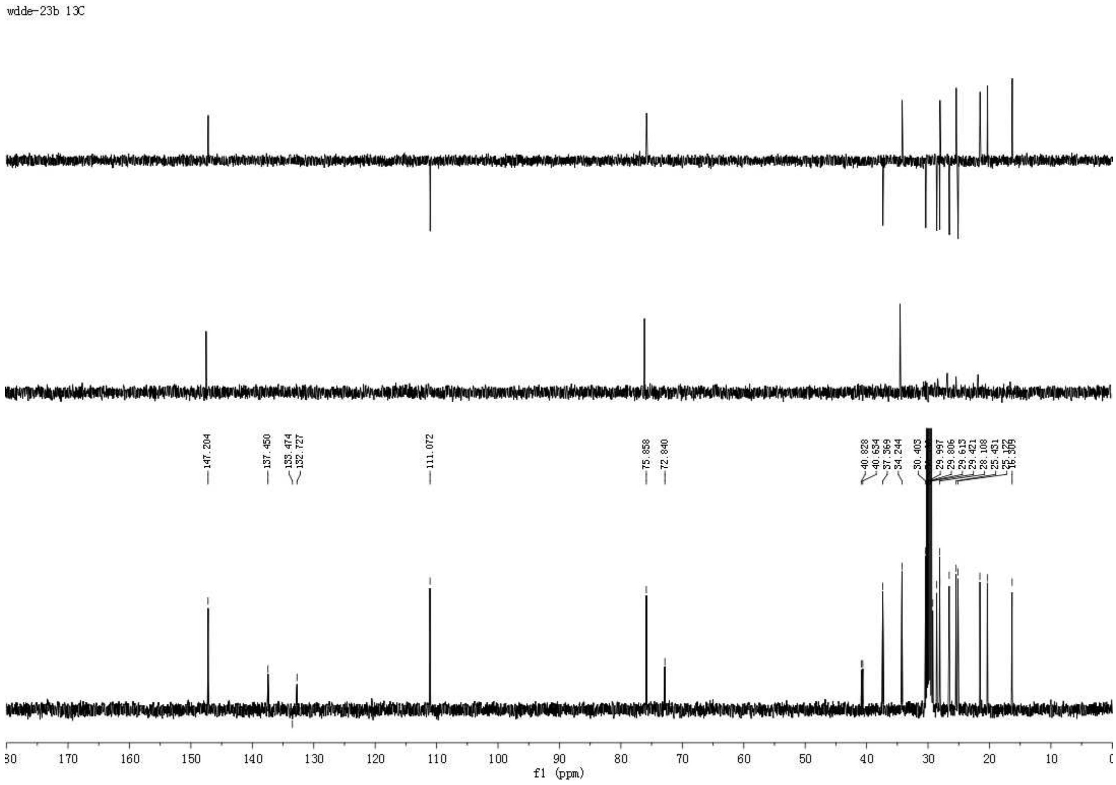

Figure 9S. HSQC of 5(10),14-halimadiene-3α,13ξ-diol (**2**)

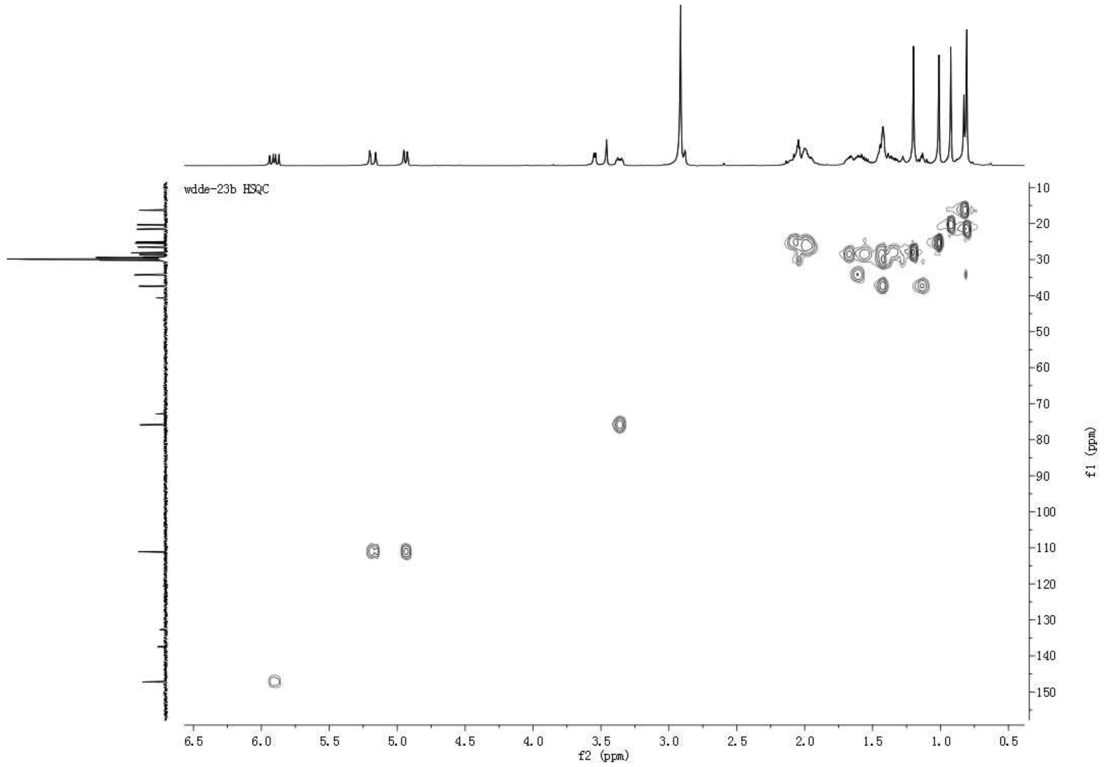

Figure 10S. HMBC of 5(10),14-halimadiene-3α,13ξ-diol (**2**)

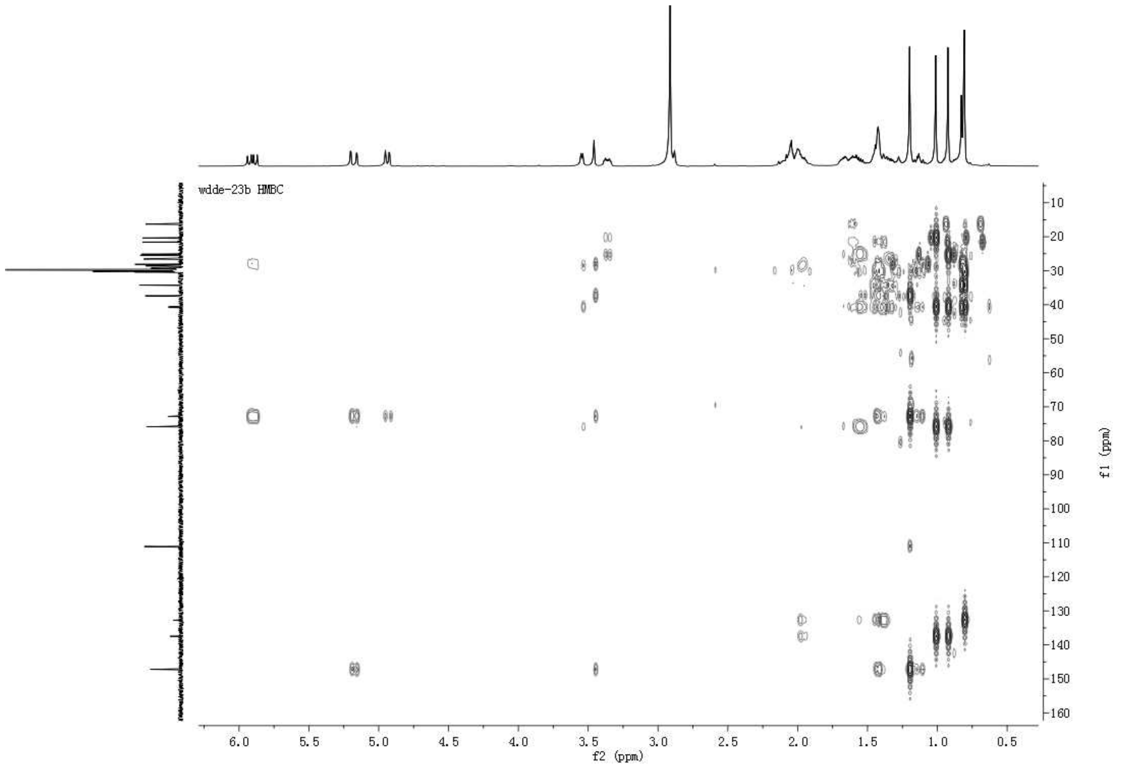

Figure 11S. ROESY of 5(10),14-halimadiene-3α,13ξ-diol (**2**)

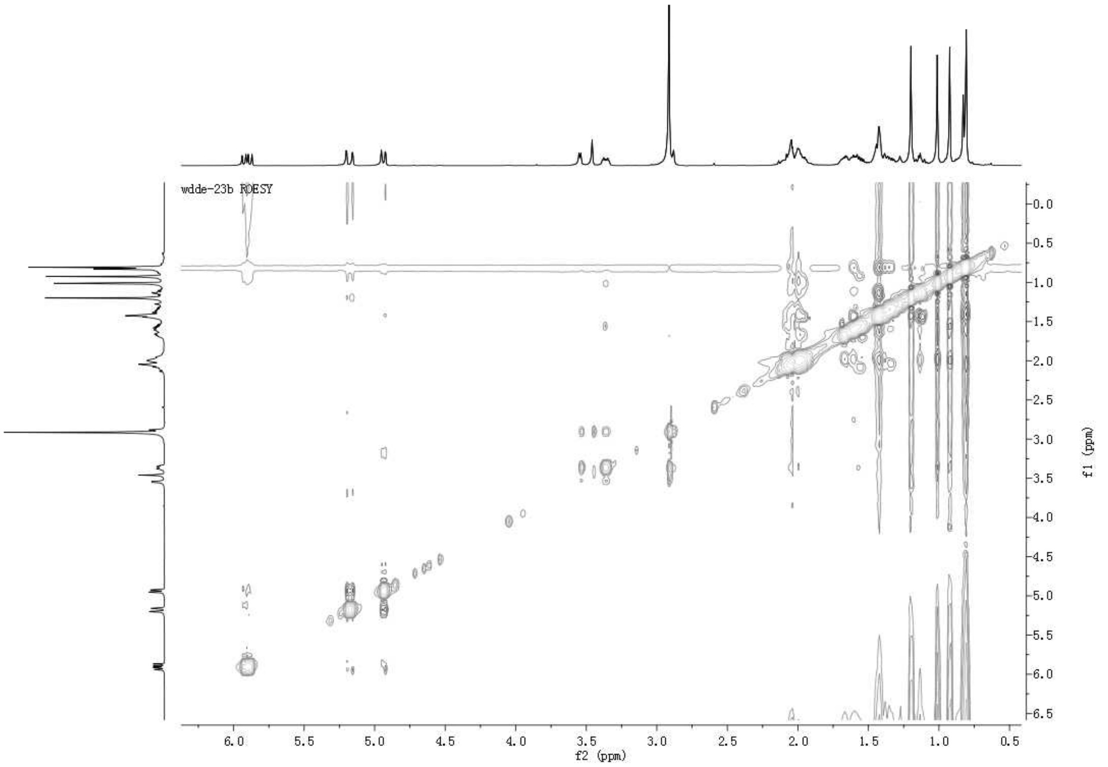

Figure 12S. HREIMS of 5(10),14-halimadiene-3α,13ξ-diol (**2**)

Elemental Composition Report — **Page 1**

Single Mass Analysis

Tolerance = 10.0 PPM / DBE: min = -10.0, max = 120.0

Selected filters: None

Monoisotopic Mass, Odd and Even Electron Ions

15 formula(e) evaluated with 1 results within limits (up to 51 closest results for each mass)

Elements Used:

C: 0-200 H: 0-400 O: 2-4

wdde23b
16:56:54 20-Nov-2012
Voltage EI+

KIB
M121122EA-11AFAMM 14 (1.286)

Autospec Premier
P776
45.7

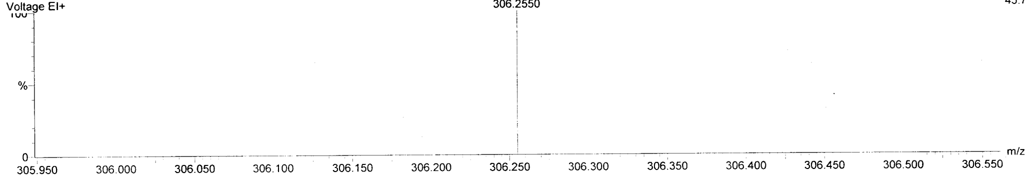

Minimum:				-10.0		
Maximum:		100.0	10.0	120.0		
Mass	Calc. Mass	mDa	PPM	DBE	i-FIT	Formula
306.2550	306.2559	-0.9	-2.9	4.0	5546038.5	C20 H34 O2

Figure 13S. ^{1}H NMR of 12-(3-methyl-furan)-labd-8(17)-en-19-oic acid (**3**)

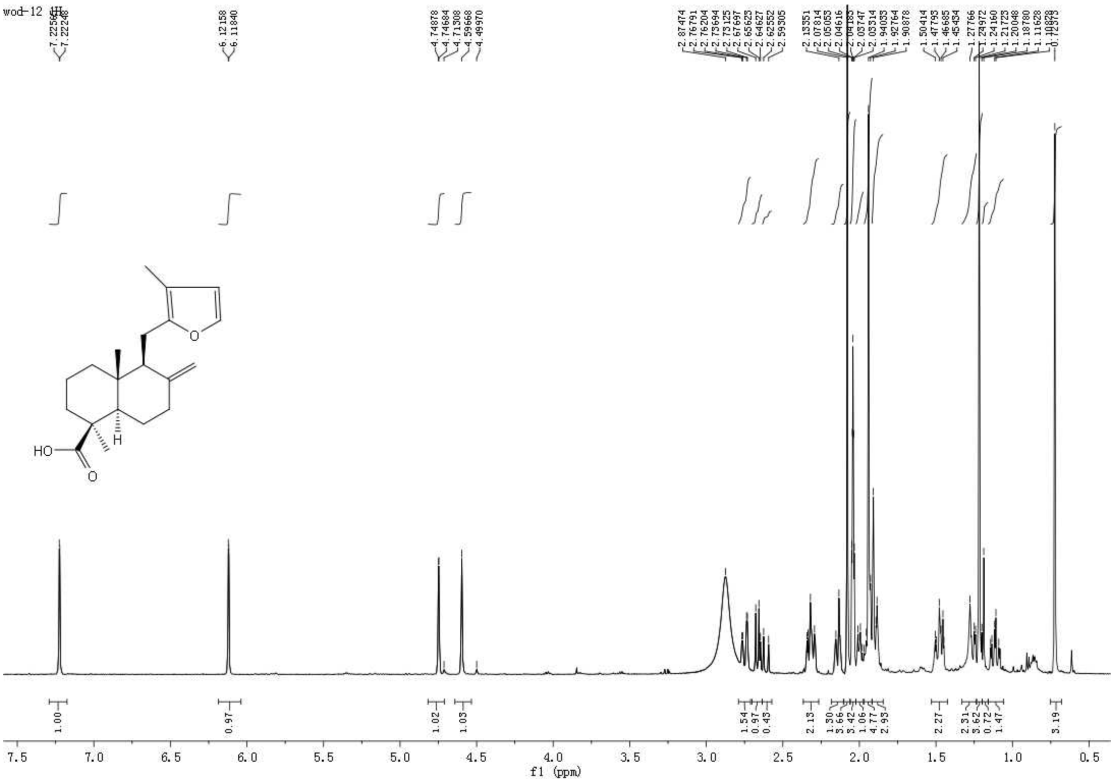

Figure 14S. ^{13}C NMR and DEPT of 12-(3-methyl-furan)-labd-8(17)-en-19-oic acid (**3**)

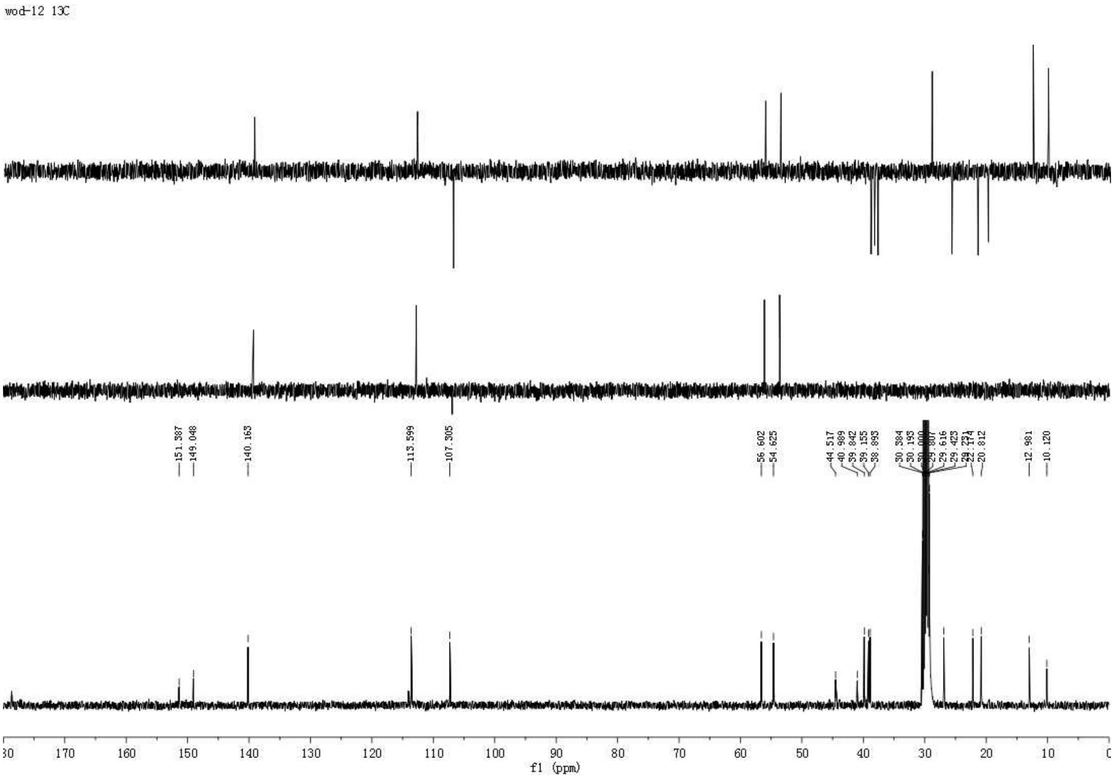

Figure 15S. HSQC of 12-(3-methyl-furan)-labd-8(17)-en-19-oic acid (**3**)

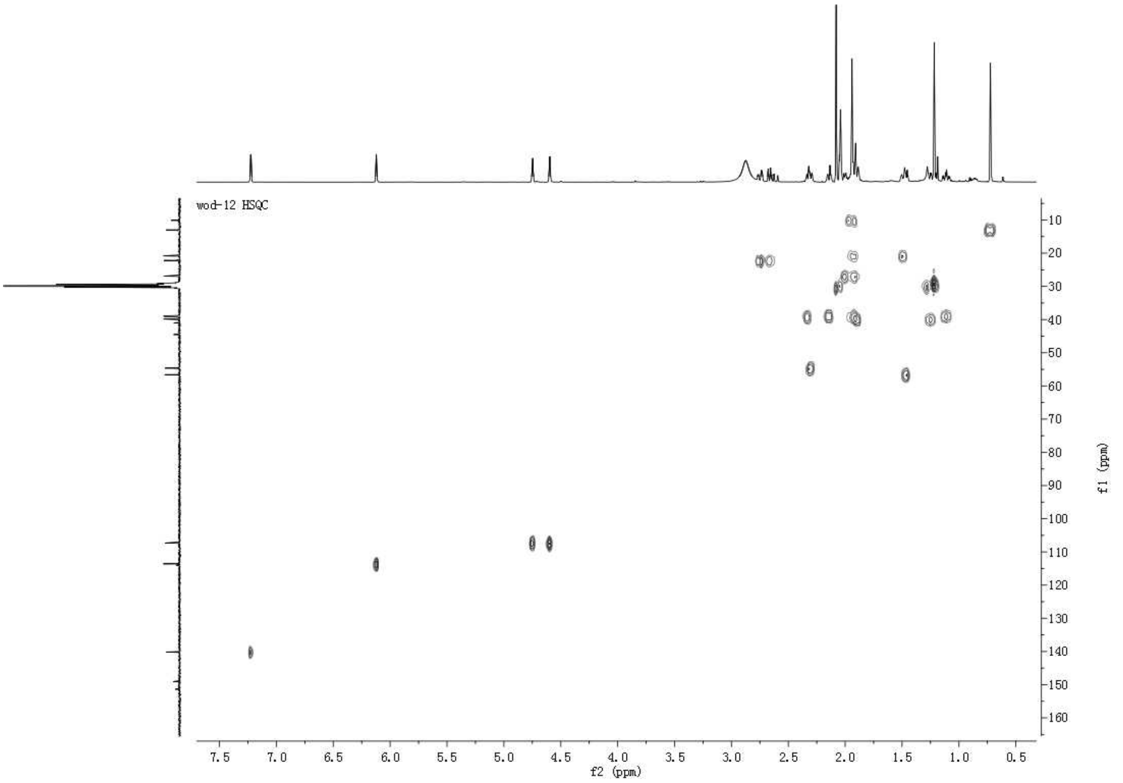

Figure 16S. HMBC of 12-(3-methyl-furan)-labd-8(17)-en-19-oic acid (**3**)

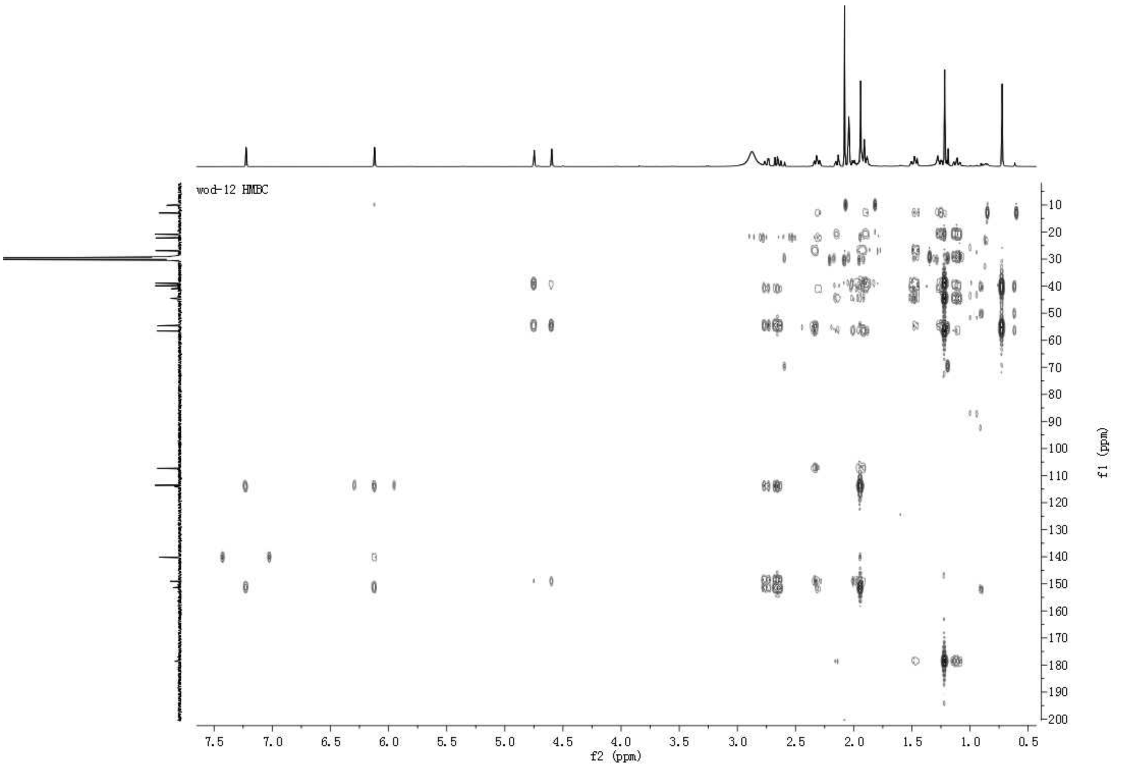

Figure 17S. ROESY of 12-(3-methyl-furan)-labd-8(17)-en-19-oic acid (**3**)

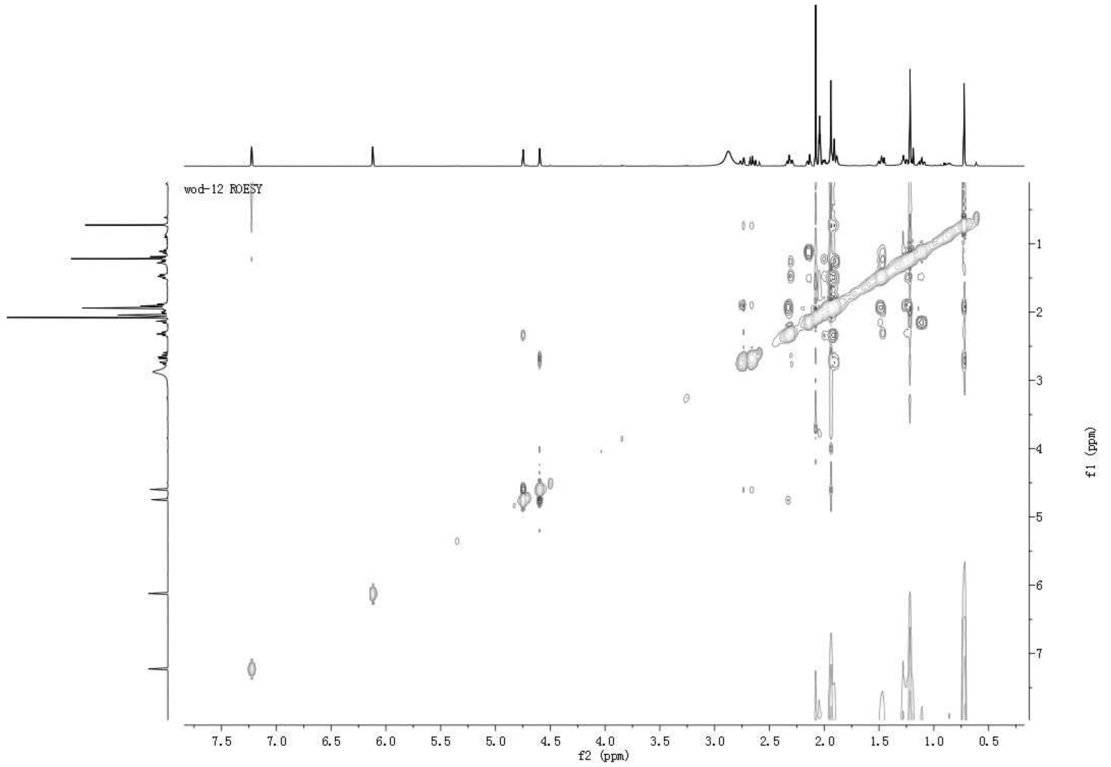

Figure 18S. HREIMS of 12-(3-methyl-furan)-labd-8(17)-en-19-oic acid (**3**)

Elemental Composition Report **Page 1**

Single Mass Analysis
Tolerance = 10.0 PPM / DBE: min = -10.0, max = 120.0
Selected filters: None

Monoisotopic Mass, Odd and Even Electron Ions
16 formula(e) evaluated with 1 results within limits (up to 51 closest results for each mass)
Elements Used:
C: 0-200 H: 0-400 O: 2-4

wod-12 KIB Autospec Premier
10:20:39 23-Nov-2012 M121123EA-09AFAMM 26 (2.388) P776
Voltage EI+ 316.2039 17.2

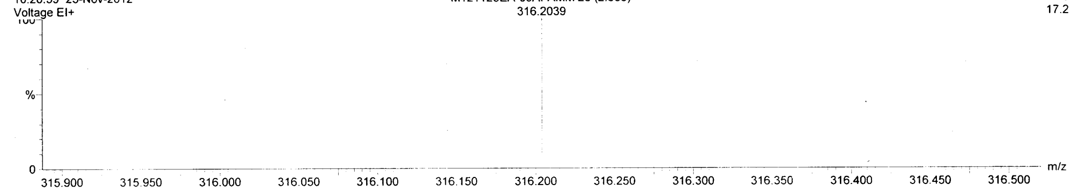

Minimum:				-10.0		
Maximum:		100.0	10.0	120.0		
Mass	Calc. Mass	mDa	PPM	DBE	i-FIT	Formula
316.2039	316.2038	0.1	0.3	7.0	5546027.5	C20 H28 O3
